# COMPARISON OF CAREGIVERS FROM DIVERSE COMMUNITIES WHO IMMIGRATED TO OR WERE BORN IN THE US

**DOI:** 10.1093/geroni/igz038.3465

**Published:** 2019-11-08

**Authors:** Lauren Stratton, David Bass, Rachel Schaffer, Sara Powers, Ocean Le, Jenna McDavid

**Affiliations:** 1 Iowa State University, Ames, Iowa, United States; 2 Benjamin Rose Institute on Aging, Cleveland, Ohio, United States; 3 Diverse Elders Coalition, New York, New York, United States

## Abstract

The Diverse Elders Coalition, in partnership with its six member organizations and the Benjamin Rose Institute on Aging, completed a national survey of 840 family and friend caregivers from diverse racial, ethnic, and sexual orientation communities to understand their unique caregiving issues and challenges. Data from a subsample of 369 caregivers identifying as Hispanic/Latino, Asian, Southeast Asian or multiple ethnicities were analyzed to understand similarities and differences between caregivers born in the US and who immigrated to the US. The Stress Process Conceptual Model guided selection of characteristics used for comparative analysis. Results of logistic regression revealed that caregivers born in the US were younger (B=-.08, p<.001), had higher educational degrees (B=.42, p<.001), and higher incomes (B=.34, p=.002). They assisted care receivers with more health-related tasks (B=.27, p=.013), but fewer culture-related tasks (B=-.51, p=.002); reported higher levels of strain in their relationship with care receivers (B=.66, p=.038); and were less satisfied with the quality of care receivers’ healthcare (B=-.62, p=.042). In terms of reasons for being a caregiver, there were no significant differences in cultural commitment to caring for older family members, however those born in the US were more likely to report providing care because it was more convenient for them than for other family and friends (B=.99, p=.002). Understanding the needs of diverse caregivers has implications for healthcare and service providers, such as providing training on diverse needs. Additionally, the differences between US born and immigrant caregivers highlights implications on the dynamic between caregivers and their care receiver.

